# Clinical and Microbiological Profiles of Urinary Tract Infections in Febrile Children Aged Six Months to Five Years Attending a Tertiary Care Hospital in India

**DOI:** 10.7759/cureus.51903

**Published:** 2024-01-08

**Authors:** Anusree Krishna Mandal, Jadab Kumar Jana, Yendrila Chatterjee, Mohan Pradhan, Dipti Mahata, Md Suhail A Mallick

**Affiliations:** 1 Pediatrics, Bankura Sammilani Medical College and Hospital, Bankura, IND; 2 Cardiology, Bankura Sammilani Medical College and Hospital, Bankura, IND; 3 General Surgery, Bankura Sammilani Medical College and Hospital, Bankura, IND

**Keywords:** uti recurrence, renal system, fever without focus, children, antibiotic sensitivity and resistance

## Abstract

Background

Urinary tract infection (UTI) is one of the most common causes of fever in the pediatric age group. The study was designed to study the clinical profile, etiologic microorganisms, and antibiogram patterns.

Methods

The present study is a hospital-based cross-sectional study done over a study period of one and a half years.

Results

Females comprised a higher proportion of the study subjects. Increased urinary frequency and urgency were the most prevalent presenting symptoms in the study population, seen in 39 (39%) and 20 (20%) of the children, respectively. *Escherichia coli* was found to be the most common causative organism in 45 (45%) children followed by Klebsiella in 22 (22%) children. The organisms showed maximum sensitivity to linezolid (88%) followed by levofloxacin(78%), and piperacillin-tazobactam(76%). Cotrimoxazole(16%) and cefixime(9%) showed the maximum resistance. The outcome was favorable for the majority of the patients treated at par with the antibiogram. Eleven (11%) of the children were found to have anatomical abnormalities in their genitourinary system, and it was found to be significantly associated with recurrence (P value=0.05).

Conclusions

UTI as one of the leading causes of fever and has to be dealt with a high index of suspicion while evaluating for cases of fever without a focus on children. The antibiogram of the underlying organisms needs to be followed while treating cases of UTI to ensure prompt recovery and avoid the emergence of antimicrobial resistance. This also highlights the need for periodic surveillance of the local prevalence of organisms and their antimicrobial susceptibilities to tailor proper management. Children with anatomical abnormalities in their renal system need to be followed up carefully for chances of recurrence.

## Introduction

Urinary tract infection (UTI) associated with fever is a very common ailment in children below five years of age. However, UTI as a cause of fever in this age range receives little attention and can also manifest as a fever of unknown origin [1]. Furthermore, UTI is one of the most prevalent causes of fever in children and can have serious long-term consequences if left untreated. Hence, it is essential to identify UTIs in febrile children and initiate prompt therapy; otherwise, progressive renal damage from unrecognized pyelonephritis in children may gradually lead to hypertension and chronic renal failure [2]. The presenting signs and symptoms of UTI in childhood can be mostly non-specific, especially among infants. Owing to non-specific presentation and a multitude of etiologies of fever, pediatricians are frequently left with the dilemma of whether to evaluate the child for an underlying UTI. The common causes of UTI in children are mostly bacterial like *Escherichia coli (E. coli).* Others like *Pseudomonas *and *Proteus* are prevalent in the presence of an underlying obstruction or instrumentation. Organisms like* Klebsiella, Enterococci, Staphylococcus saprophyticus*, and *Candida albicans* may also be responsible [3]. Cystitis with Candida may occur in patients with urinary catheterization following antibiotic therapy [4]. UTI can also be caused by viruses as part of the non-bacterial UTI spectrum affecting children. Examples are adenovirus, influenza, cytomegalovirus, herpes simplex viruses, and polyomavirus. They may cause irritative voiding symptoms, hemorrhagic cystitis, and even urinary reflux or retention. However, these forms of cystitis are mostly self-limited unless the child is immunosuppressed [5].

The clinical presentation of UTI in infants and young children can vary from occult and undiagnosed fever to gastrointestinal manifestations as well as upper and lower urinary tract symptoms whereas, in older children, more specific symptoms referring to the urinary tract may be observed [6]. Susceptibility patterns of the bacterial isolates vary with geographic region and act as a reference for guiding the empirical therapy. The etiology of pediatric UTI and the antibiotic susceptibility of urinary pathogens in both the community and hospitals have been changing, and drug resistance has become a major problem [7-11].

## Materials and methods

Objective

The present study aimed to describe the clinical profile of UTI in febrile children aged six months to five years along with documenting the demography, bacteriological profile, antibiogram, and radiographic patterns of the renal system in the study population.

Study area

The study was carried out in Bankura Sammilani Medical College and Hospital's (BSMCH) pediatric in-patient and outpatient department (OPD). 

Study design

It was a hospital-based cross-sectional study carried out from February 2021 to July 2022.

Study population

Inclusion Criteria

Children aged >=6 months and <=5 years attending the study area during the study period, with a fever of axillary temperature exceeding 100 degrees Fahrenheit and diagnosed with UTI by significant bacteriuria with a colony count of >10^5/ml of a single species in a clean catch sample, were included. The urine may be obtained by suprapubic aspiration or urethral catheterization in children below two years. Any colonies on suprapubic aspiration and >50,000/ml on urethral catheterization were considered significant.

Exclusion Criteria

Those who were not willing to take part in this study or any child who received antibiotics 48 hours prior to admission or attending the OPD were excluded.

Sample size determination

The sample size was calculated using the following formula:

N=(Zα)2 x p (100 -p)/ L2

Where, Zα = 1.96 at 95% confidence interval, p = prevalence of event of interest = 6.36% as per previous research [12], L = absolute error around the reported prevalence. Here it is assumed to be 5 (absolute). Therefore, putting these values into the formula, the estimated sample size would be n = (3.84*6.36*93.64) = 91.4=91. Considering a 10% non-response rate, the revised sample size will be =91+10% of 91=100. It was also determined that the data collection will be carried out based on a two days per week schedule. These two days of each were chosen at the beginning of the concerned week and a consecutive of 100 total febrile patients was taken.

Study tools and techniques

These included an interview of either the parents of patients or caregivers using a pre-tested and pre-designed interviewer-administered questionnaire, a clinical examination of the patients, and a review of medical records.

Data analysis

Quantitative variables were described by mean, standard deviation, and range. The qualitative variables were presented by proportion. Data display was achieved using tables and charts. All the data collected was entered in a Microsoft Excel spreadsheet (Microsoft Corporation, Redmond, WA, USA). The statistical analysis was carried out using available standard statistical software (SPSS version 22; IBM Corp, Armonk, NY, USA) using Fisher’s exact test and the chi-square test, wherever applicable. The level of significance was expressed as p-value <0.05.

Ethical consideration and consent

The Institutional Ethics Committee of Bankura Sammilani Medical College and Hospital gave its clearance before the initiation of the study (memo number: BSMC/Aca:160, dated: 19.01.2021).

## Results

The majority of the study population was female, with the female-to-male ratio being 1.2. Most of the children were in the age group of one to three years(47%). The mean age group of the total population was two years and six months. A higher proportion of the study population hailed from rural areas with the ratio of rural to urban being 6.6. The patients presented with a myriad of signs and symptoms of which the most common was that of increased frequency of micturition. The spectrum of clinical features in the study subjects is shown in Table 1.

**Table 1:** Spectrum of clinical features in the study subjects

Clinical features	No of patients, n(%)
Increased frequency of micturition	39 (39)
Urgency	20 (20)
Crying during micturition	28 (28)
Vomiting	32 (32)
Foul-smelling urine	21 (21)
Abdominal/flank pain	44 (44)
High BP	10 (10)

**Figure 1:**
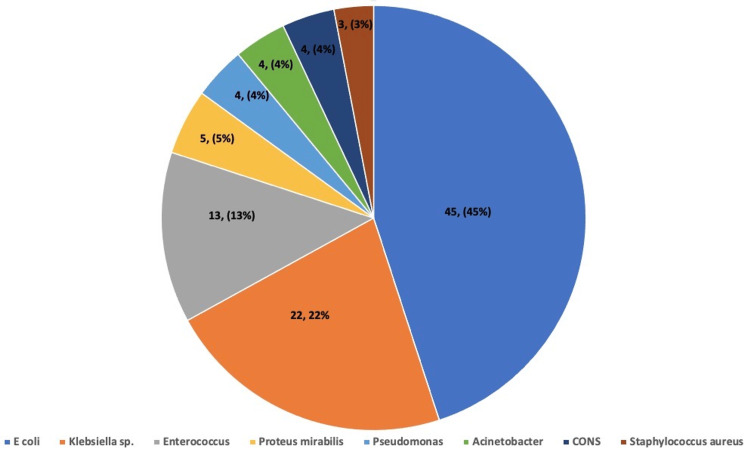
Urine culture growth patterns among the cases

**Figure 2:**
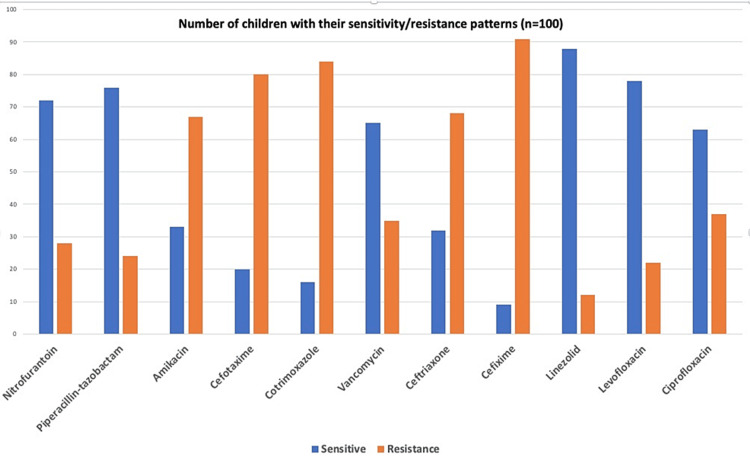
Sensitivity and resistance patterns among microorganisms

Urinalysis revealed pyuria in 25 (25%) patients and RBCs in 18 (18%). There was also proteinuria (1+ or 2+) in 23 (23%) patients. In the majority of the patients (45%), growth of *Escherichia (E.) coli *was found followed by a growth of *Klebsiella *(22%), and *Enterococcus *(13%). *Proteus mirabilis *was found in five (5%) of the patients followed by growth of *Pseudomonas, Acinetobacter, *and coagulase-negative *Staphylococci *(CoNS) in 4% of the patients each. The least common organism found was *Staphylococcus aureus* found in 3 (3%) of the patients. The most common antibiotic found to have sensitivity in the microorganisms involved was linezolid in 88 patients (88%) followed by levofloxacin (78%) and piperacillin-tazobactam (76%). Other commonly used antibiotics like ceftriaxone and vancomycin showed a sensitivity pattern in 32% and 65% of the cases, respectively. The antibiotics that exhibited the maximum resistance and least sensitivity were found to be cotrimoxazole (16%) and cefixime (9%). The patients were treated in keeping with the sensitivity patterns of the etiologic microorganisms. The majority of the patients (45%) were treated with piperacillin-tazobactam followed by amikacin and nitrofurantoin in 15 (15%) patients each. Vancomycin was used in 13 (13%) of the patients and levofloxacin was used in 4 (4%) of the patients followed by cefotaxime in 3 (3%) patients. The least common antibiotics were imipenem and vancomycin, which were used to treat 2 (2%) and 1 (1%) patients, respectively. The urine culture growth patterns among the cases are demonstrated in Figure 1, whereas the sensitivity and resistance patterns among the microorganisms are shown in Figure 2.

Fifty-four (54%) patients required a hospital stay of 7 days for full recovery. Twenty-four(24%) patients stayed for 10 days followed by 17 (17%) patients who stayed for 5 days. The maximum duration of hospital stay for full recovery was 14 days, which was seen for 5 (5%) patients. Most of the patients (88%) were successfully treated and were discharged after a full recovery. Eleven (11%) of the patients were found to have a recurrence of their UTI. There was a single case fatality, making the mortality of the study 1%. The distribution of the outcomes of the patients is shown in Table 2.

**Table 2:** Distribution of the outcome of the study subjects

Outcome	No. of patients, n(%)
Recovered and discharged	88 (88)
Recurrence	11 (11)
Died	1 (1%)

Ultrasonography of the kidney, ureters, and bladder (USG KUB) turned out to be normal in the majority of patients (65%). The majority of the patients showed no abnormality on USG KUB. Twenty-six (26%) of them had cystitis on USG KUB. Two (2%) of them had ectopic kidneys. Four (4%) had either unilateral or bilateral hydronephrosis. Two (2%) of them had findings suggestive of vesicoureteral reflux (VUR) and in one (1%) bladder calculi can be seen. The anatomical abnormalities noted can be a possible risk factor for the UTI itself and its subsequent recurrences.

Table 3 shows the distribution of the USG KUB findings of the patients.

**Table 3:** Distribution of the USG KUB findings of the patients USG KUB: ultrasonography of the kidney, ureters, and bladder

USG KUB	Total, n(%)
No significant abnormality	65 (65%)
Cystitis	26 (26%)
Ectopic kidney	2 (2%)
Left-sided hydronephrosis	1 (1%)
Right-sided hydronephrosis	1 (1%)
Bilateral (B/L) hydronephrosis	1 (1%)
B/L hydronephrosis with thickened bladder wall	1 (1%)
Dilatation of the pelvic-calyces s/o vesicoureteral reflux (VUR)	1 (1%)
Dilatation of B/L pelvic-calyces s/o VUR	1 (1%)
Bladder calculi	1 (1%)

An association was found between anatomical abnormalities on USG KUB and recurrence in patients (Table 4).

**Table 4:** Association between anatomical abnormalities on USG KUB and recurrence of UTI USG KUB: ultrasonography of the kidney, ureters, and bladder; UTI: urinary tract infection

Presence of recurrence	Total, n(%)	Anatomical abnormalities on USG		P value=0.0000001 (Mid-P exact test)
		Present	Absent	
Yes	9 (9)	8	1	
No	91 (91)	3	88	
Total	100 (100)	11	89	

In the present study, 73% of children (n = 8) presented, with recurrence among those who had the presence of anatomical abnormalities. Therefore, anatomical abnormalities present on USG are significantly associated with recurrence (p-value < 0.05).

## Discussion

UTIs are one of the most common and serious infections found in children. They are also a serious cause of morbidity and may lead to permanent sequelae, which include diseases like hypertension and renal failure. Early diagnosis of UTIs is essential, as it aids in prompt treatment and ensures proper evaluation and follow-up of the child. Excluding UTI is of utmost importance in a febrile child to decrease the morbidity burden of the patients and any possible long-term sequelae.

Demographics

In the present study, out of 100 children, 56 were female and 44 were male. The ratio of females to males was 1.2. In a study done by Jitendranath et al., females were also shown to be predominantly affected [13]. In the present study, 22 children were in the age group of 6 months to 1 year, 47 in the age group of 1-3 years, and 31 in the age group of 3-5 years. The mean age group of the total population was two years and six months. Among the 100 children included in the present study, the majority of the children were in the age group of one to three years (42%). A retrospective study done by Garout et al. showed a mean age of 15 months with a standard deviation of 19.86 [14].

Clinical symptoms

All of the patients had a myriad of clinical symptoms along with pre-existing fever. Among the 100 patients, 39 (39%) presented with increased frequency of micturition, 28 (28%) had complaints of crying during micturition, and 32 (32%) had complaints of vomiting. Furthermore, 21 (21%) presented with a complaint of foul-smelling urine, and 44 (44%) presented with a complaint of abdominal or flank pain. Six had coexisting lower limb neurological deficits (6%). Further possible complications noted over a follow-up period of 1 year, included hypertension in 10 of them (10%) and low BP or hypotension in 6 (6%) of them.

In a study done by Medina-Bambardo D, et al., it was seen that almost 90% of the patients presented with complaints of urinary tract symptoms like increased frequency of micturition, painful micturition, or urgency, which is more than that seen in the study [15].

Urinalysis

In the present study on 100 patients, 25 (25%) showed plenty of pus cells per hpf. The majority of them (82%) did not show any RBCs on microscopic examination, whereas RBCs were seen in the remaining patients (18%). The majority of the patients with UTI showed no proteinuria but 20 of them (20%) either had trace or + protein and 14 of them (14%) showed 2+ proteinuria. In the majority of the patients (45%), growth of* E. coli* was found, followed by growth of *Klebsiella *(22%) and *Enterococcus *(13%). *Proteus mirabilis* was found in 5 (5%) of the patients followed by growth of *Pseudomonas, Acinetobacte*r, and CoNS in 4% of the patients each. The findings are comparable to other studies. In a study done by Jitendranath et al., *E. coli *was found to be the most common isolate followed by *Klebsiella *[13], which is comparable to the present study.

The most common antibiotic found to have sensitivity in the microorganisms involved was linezolid in 88 patients (88%) followed by levofloxacin (78%) and piperacillin-tazobactam(76%). Other commonly used antibiotics like vancomycin and ceftriaxone showed a sensitivity pattern in 65% and 32% of the cases, respectively. The antibiotics that exhibited the maximum resistance and least sensitivity were found to be cotrimoxazole (16%) and cefixime (9%). The findings are somehow different from other similar studies. A study done by Badhan et al showed the most active antibiotics to be nitrofurantoin, cefotaxime, amikacin, and cotrimoxazole [16], whereas another study done by Singh et al. showed maximum sensitivity to amikacin and vancomycin [17]. This could be due to different organism sensitivity and resistance patterns, depending on the geographic distribution and varied antibiotic protocols.

USG findings

The majority of the patients showed no abnormality on USG KUB. Twenty-six (26%) of them had cystitis on USG KUB. Two (2%) of them had ectopic kidneys. Four (4%) had either unilateral or bilateral hydronephrosis. Two (2%) of them had findings suggestive of VUR and in one (1%), bladder calculi can be seen. The anatomical abnormalities noted can be a possible risk factor for the UTI itself and its subsequent recurrences. In a study done by Ahmadzadeh et al. [18], findings of VUR were seen in 40% of the UTI patients and 20% had other associated urinary tract abnormalities.

Limitations of the study

The present study focuses only on UTI as the underlying cause of fever. In the study, there remained a chance of contamination of samples, as collection is relatively difficult in children. Also, in our setting, there was a lack of availability of investigations like voiding cystourethrogram and dimercaptosuccinic acid (DMSA) scan for further evaluation.

## Conclusions

In the present study, UTI was found to be more prevalent in female children. The majority of the children belonged to the age group of one to three years. The major presenting clinical symptoms were increased frequency of micturition, crying during micturition, urgency, lower abdominal or flank pain, foul-smelling urine, and vomiting. Urinalysis showed plenty of pus cells per hpf in most of the patients. A few of them also had RBCs on microscopic examination and some had traces of 2+ proteinuria. The most common organism growth was that of* E. coli *followed by *Klebsiella* sp., *Enterococcus*, *Proteus mirabilis,* and *Pseudomonas*. The most commonly active antibiotics were found to be linezolid and levofloxacin, followed by piperacillin-tazobactam, and vancomycin. USG KUB findings were most commonly suggestive of cystitis followed by pre-existing anomalies like hydronephrosis, VUR, and ectopic kidney. Anatomical abnormalities on USG were found to be significantly associated with a recurrence of UTI in the children. The majority of the patients required a hospital stay of seven days for full recovery. The minimum and maximum duration of hospital stay for full recovery were 5 and 14 days, respectively. The majority of the patients were successfully treated and discharged after full recovery, with some of them having a recurrence of their UTI. There was a single case fatality in the present study. All of these findings are in accordance with many previous studies and, hence, reliable.
